# Medical and Physical Intelligence

**Published:** 1822-05

**Authors:** 


### Medical and Physical Intelligence.

### 1. *Arracacha.*

THE root of this plant, from Santa Fè, is expected soon by one of our first naturalists. It grows in a climate of the temperature of England; and, from the account given of it in the Annals of Botany, it promises to equal the potato, although it is not of the family of *solanum*, but probably a *pastinaca*. We cannot have too many good things of this sort; although, it must be owned, we do not yet know the extent of the value of the *solanum tuberosum*.—*Ed. Med. Jour.*

### 2. *Use of the Piper Cubeba in Leucorrhœa.*

Dr. James Orr, of Edinburgh, has communicated to Dr. Duncan, jun. four cases of leucorrhœa cured by cubebs. In the first case, the patient had laboured under the disease for seven months. In the second, the complaint had lasted nearly eight years; in the third, it was of two years' standing; and in the fourth, the discharge had resisted the use of several remedies for six months. In the first three cases, the disease succeeded to abortion. The pepper was administered either in water or milk, or made into an electuary with treacle. The dose was from one drachm and a half and upwards, three times a-day, but never exceeded three drachms.

### 3. *New Metal.*

Counsellor Giesse, of Dorpat, has communicated to the world the discovery of what he at present thinks to be a new metal, extracted from the residue of English sulphuric acid, on distilling it to dryness. One variety left, out of sixteen ounces, 9½ grains of a white residuum, in which there was no sulphate of lead. It changed colour several times during the experiments made upon it, and he thinks it was formed of the sulphur employed in manufacturing the acid. It is susceptible of oxidation, and its alkaline combinations form double salts with acids. Still the professor's details are judged, on the whole, to be inconclusive.—*Philos. Mag.*

### 4. Professor Kuhn's *Edition of the Works of* Galen.

Κλαυδιον Γαληνου Απαντα. *Claudii Galeni Opera omnia.* Edit. cur. D. Car. Gottlieb Kühn. T. II. Lipsiæ, 1821.

Every lover of Greek literature will hail with pleasure the appearance of this second volume of the "*Medicorum Græcorum Opera quæ extant*," so speedily after the publication of the first. This activity induces us to augur well respecting the completion of the work, and to cherish an earnest hope that the learned editor, though advanced in years, may be spared to complete this great undertaking. We sincerely hope our countrymen will not be backward in patronizing this work, but that they will give their assistance liberally; particularly as copies may be readily procured through the medium of the foreign medical booksellers in Edinburgh or London. The contents of this volume are as follow:—1. De naturalibus facultatibus, pp. 1 to 214. 2. De anatomicis administrationibus, pp. 215-731. 3. De ossibus ad tirones, pp. 732-778. 4. De venarum arteriarumque dissectione, pp. 779-830. 5. De nervorum dissectione, pp. 831-856. 6. De instrumento odoratus, pp. 857-886. 7. De uteri dissectione, pp. 887-908.—*Ed. Med. Journal.*

### 5. *On the Setting of Surgical Instruments.*

Mr. Reveley recommends the use of soap instead of oil for the setting of razors and other cutting instruments on a hone. His own words are:—" Not having any oil to set my razor, it occurred to me to try the soap I was washing with, called palm-soap, and I found it so completely to answer my purpose, that I have constantly used it ever since instead of oil, both for razors and penknives. It sets quicker, gives a good edge, and removes notches with great facility: it is a more cleanly material, oil being liable to drop on, and soil, any thing it comes in contact with; dust will frequently get into oil, which will spoil the edge, and in such case it must be changed. It is as cheap or cheaper than oil, a small square of palm-soap costing only threepence, which will last for a great length of time. The operation is performed as follows: Having first cleaned your hone with a sponge, soap, and water, wipe it dry; then dip the soap in some clean soft water, and, wetting also the hone, rub the square of soap lightly over it until the surface is thinly covered all over; then proceed to set in the usual way, keeping the soap sufficiently moist, and adding from time to time a little more soap and water, if it should be necessary. Observe the soap is clean and free from dust before you rub it on the hone: if it should not be so, it is easily washed clean. Strop the razor after setting, and also again when you put it by, and sponge the hone when you have done with it."

Many instrument-makers and others have tried Mr. Reveley's method of setting instruments, and speak in high commendation of it. Among them are Messrs. West, Pepys, Long, and Frewen.—*Trans. Soc. Art.* xxix. 137.

### 6. *Preparation of Ethiop's Mineral.*

Dr. Taddei recommends the following process for the preparation of this substance, as being one which effects the combination immediately, and in a more perfect manner than that generally employed.—Put one part of sulphuret of potash into a mortar, with three or four parts of running mercury; triturate together, adding a little water by degrees, until the whole is reduced to a homogeneous black paste; then add flowers of sulphur, in equal quantity to the mercury employed, and mix the whole by a short trituration. Then wash the whole; and filter with repeated portions of water, till all the alkaline sulphuret is removed.

Ethiops thus prepared is not of the black colour of that obtained by simple trituration, but it is a more perfect combination. Dr. Taddei says, that the addition of a little sulphuret of potassa to the mixture of sulphur and mercury, does not do away with a long trituration, but that, proceeding as above, the substance is prepared instantly.—*Giornale de Fisica*, iv. 12.

### 7. *Inoculation of the Human Subject with the Matter of Glanders.*

A horse belonging to the Badsworth Hunt having died of the glanders, the dog-feeder to the hounds was ordered to cut up the carcase, in doing which he accidentally wounded his hand, and thereby inoculated his own system with the poisonous matter from the dead horse. In a few days he betrayed all the symptoms which are at first shown in the horses beginning in the above disease. He gradually became worse; and at the end of the week he died raving mad, labouring under a confirmed complaint of glanders. The rest of the paper details the distressed state of his family, (a wife and ten children,) and solicits aid.—(J. MUSCROFT, *Surgeon, Pontecraft; June* 4, 1821.)

8. *Solution of Oxide of Copper in Ammonia.*

M. BERZELIUS, in his description of a method of analyzing the ores of nickel, says, that he had not been able to determine whether oxide of copper is soluble in ammonia or not. "It is certain that all those solutions, which are generally regarded as oxide of copper in ammonia, are double salts, with excess of base. I digested oxide of copper in concentrated ammonia, for eight days, in a stopped bottle. The solution became of a light-blue colour in forty-eight hours, and it did not afterwards increase. A drop of carbonate of ammonia, let fall into the liquor, immediately dissolved a part of the oxide, and made the lower stratum of the liquid of a deep-blue colour."

When an ammoniacal solution of oxide of copper is mixed with caustic potash, the oxide of copper is precipitated in a few seconds; and, if the quantity of potash is sufficient, it is entirely deposited in the form of a blue hydrate, which it is very easy to wash. When well washed, it yielded blue hydrate of copper, combined with two atoms of water: it does not retain any trace of potash.—*Ann. de Chimie.*

9. *Preservation of Anatomical Specimens.*

Dr. MACARTNEY, of the Dublin University, has for some time employed a solution of alum and nitre, for the purpose of preserving anatomical preparations. He finds that it preserves the natural appearances of most parts of the body more completely than spirits or any other fluid heretofore used. The proportions of the alum and the nitre, and the strength of the solution, require to be varied according to circumstances; and, in order to thoroughly impregnate the anatomical preparations, the liquor must for some time be occasionally renewed. The solution possesses such antiseptic powers, that the most putrid and offensive animal substances are rendered perfectly free from fetor by it in a few days.

10. *Use of Sub-nitrate of Bismuth in Intermittent Fevers.*

Dr. HENKESEW, a physician at Hildesheim, has been in the habit of prescribing this remedy in agues, for several years. He considers it to be a powerful febrifuge and anti-spasmodic. He exhibits this salt in the dose of four grains, with a few grains of sugar, every two hours.

10. *On a Deposit found in the Waters of Lucca.* By Sir HUMPHREY DAVY, Bart. F.R.S. &c.

The waters of the baths at Lucca, at the spot where the temperature is the greatest,—that is to say, in what are termed the *caldi*, or hot baths,—eject, in a considerable quantity, a substance that produces a deposit of a brownish-yellow hue. Having collected various quantities of this deposit, and having submitted it to chemical experiments, I have discovered it to be a compound of oxide of iron and silica. Not having a balance sufficiently accurate, it was impossible for me to ascertain, with precision, the exact proportions. In the single experiment, however, that I made for this purpose, the oxide of iron was, to the silica, in the proportion of 4 to 3.

It is extremely probable that the oxide of iron and the silica had been dissolved together in the water, and deposited at the same time, because the silica, being separated from the oxide by means of a weak acid, it appears to resemble gelatine; and because the deposit, when examined in its natural state, was found to be uniform in its substance, even when looked at through a lens.

Although the oxide of iron, when first discovered, proves to be peroxide, it is, nevertheless, very probable that it exists in the water in the form of protoxide; or that it is converted into peroxide by the action of the air which is dissolved in the water. The probability of this opinion is further confirmed by the circumstance that the colour of the water is not changed by the addition of the triple prussiate of iron, nor by that of gallic acid; it being well known that protoxides generally have a greater deposition than peroxides.

The analogy which I established some time since, during my researches as to the decomposition of alkalies and earths, between the base of silica and that of boracic acid, and the facts described by MM. Smithson and Berzelius, furnish reasons for classing silica among the acids; and it seems probable that the oxide of iron and the silica undergo a real chemical combination in the warm water; and that they separate from it in consequence of its cooling, after issuing from the mountain.

A small portion of oxide of iron is found in the Bath waters, where likewise it is accompanied with silica; nor is it improbable that this earth is, in many cases, the cause of the oxide of iron being dissolved in the water; and these facts combined furnish us, probably, with an explanation of the manner in which ochre is generated. As to what may be the effect of the combination of oxide of iron and silica on animal bodies, it is the province of medical men to examine, and to determine upon, after long and adequate experiments.—*Ann. of Phil.*

12. *Poisoning from Tincture of Cantharides.*

Four labourers found, in a warehouse where they were working, a flask of this substance, of which they partook, supposing it to be a potable spirit. They immediately experienced a burning sensation along the course of the alimentary canal; a vomiting of blood; an impossibility to swallow; an unquenchable thirst; distention, with constant pain, in the abdomen, combined with coldness of the extremities, and a frequent and small pulse. Dr. Graaf, of Cologne, who was called to the sufferers, prescribed emulcent draughts, with camphor and nitre; leeches to the abdomen and epigastrium; emollient enemas, containing opium and camphor; the pediluvium, &c. After four days, two were out of danger. The other two continued to experience the most distressing strangury, which required emollient injections into the urinary bladder, and the following powder every two hours:—

℞. Camphoræ, gr. ij.

Fol. Uvæ Ursi, gr. j.

Gum Mimos. gr. x.—M.

Small bleedings, from time to time, were joined with this treatment; and, after the sixth day, Dr. Graaf had the satisfaction of having saved all the four.—*Hufeland's Jour.*

13. *Abdominal Hemorrhage.*

Dr. Neumann, of Berlin, was called to a woman, of a robust constitution, aged thirty-five years, who had never borne children, who had menstruated regularly, and had never experienced any ailment. She had been exposed to cold during the menstrual period, and was immediately seized with a violent pain in the abdomen. When Dr. N. saw her, she had all the symptoms of extreme loss of blood, with a distended abdomen, which was painful to the touch, and a vomiting of fecal matter. She died within a few hours. On dissection, a large quantity of blood was found in the abdomen and pelvis. The liver and spleen were soft, discoloured, and the large vessels empty. No part which evinced any breach of continuity, nor the rupture of any large vessel, could be detected. The diaphragm presented marks of inflammation. The left ovarium contained several hydatids; the right appeared like a coagulum of blood. The other organs were healthy. —*Hufeland's Jour.*

14. *Hydrophobia from a Rabid Badger.*

Two boys, aged about eight years, were bitten by an enraged badger, in the neighbourhood of Posen. One was dreadfully lacerated, and had the soft parts divided down to the bones and ligaments; the other escaped with a slight wound. Both underwent the usual precautions; but the former, from the great extent of the injuries, could only experience a partial employment of the prophylactic means. He was seized with hydrophobia on the twenty-sixth day: the latter escaped. The dissection of the badger presented nothing remarkable. The morbid inspection of the boy is not related.—*Huf. Jour.*

15. *Hydrocyanic Acid.*

Mr. Bush has been in the habit, for some time past, of using, pretty extensively, a form of medicine, the suggestion of which, he says, he owes to our Treatise on Prussic Acid, (*qy.* the second edition?) We mean the emulsion of bitter almonds, in which, as it is well known, prussic acid is naturally present. "Having witnessed," says he, "most beneficial effects from the acid, in its chemical concentrated form, in cases of pulmonary irritation and spasmodic cough, I determined to try a mixture of the bitter almond, as containing the principle in a natural state: this medicine I have given, for the last two years, with considerable success. I have the mixture prepared from a confection made in conformity to the recipe for the Confectio Amygdalæ in the Pharmacopœia Londinensis, only substituting the bitter for the sweet almond; and have given the mixture, thus prepared from a confection of bitter almonds, in doses of an ounce, or an ounce and a half every four hours: sometimes I have added to each dose a few drops of tinct. scillæ, or tinct. opii camph. I am fully convinced that this medicine is a useful addition to our pharmaceutical preparations: the ease with which it may be at all times prepared, and the certainty of its being in a state of purity, are, I consider, strong recommendations to its use. I have, in a few instances, seen it produce sickness, when given in large doses: where that has been the case, the dose has been lessened, but the medicine continued with advantage. (*Frome ; April* 3, 1822.)"

### METEOROLOGICAL JOURNAL.

By Messrs. William Harris and Co. 50, Holborn, London.

*From March* 20, 1822, *to April* 19, 1822.

| | MOON. | Rain gauge | THERM. | | | BAROM. | | DE LUC'S HYG. | | WIND. | | ATMOSPHERIC VARIATION. | | |
|---|---|---|---|---|---|---|---|---|---|---|---|---|---|---|
| | | | 9 A.M. | MAX. | MIN. | 9 A.M. | 10P.M. | 9 A.M. | 10P.M. | 9 A.M. | 10P.M. | 9 A.M. | 2 P.M. | 10P.M. |
| Mar | | | | | | | | | | | | | | |
| 20 | | | 53 | 61 | 46 | 30.32 | 30.30 | 67 | 67 | W | WNW | Fair | | |
| 21 | | | 50 | 56 | 43 | 30.25 | 30.13 | 63 | 61 | W var. | W var. | Fair | Fine | Fair |
| 22 | | | 48 | 56 | 40 | 30.34 | 30.34 | 50 | 55 | NNW | W | VeryFine | Fine | |
| 23 | ● | | 46 | 61 | 47 | 30.24 | 29.93 | 48 | 55 | W | WSW | VeryFine | | Fair |
| 24 | | | 50 | 60 | 38 | 29.61 | 29.78 | 62 | 58 | WNW | W | Cloudy | Fine | |
| 25 | | .11 | 44 | 58 | 39 | 29.80 | 30.00 | 56 | 60 | WNW | W | Fair | Rain | Fine |
| 26 | | | 44 | 60 | 37 | 30.06 | 30.21 | 63 | 65 | W | W | Foggy | Fair | |
| 27 | | | 45 | 57 | 48 | 30.17 | 30.06 | 61 | 60 | SW | SSW | Foggy | Fine | |
| 28 | | .07 | 53 | 67 | 46 | 29.96 | 30.11 | 56 | 61 | SSW | WNW | Fine | | Sho'ry |
| 29 | ☾ | | 52 | 63 | 48 | 30.46 | 30.27 | 58 | 49 | WNW | SW | Fine | | Fair |
| 30 | | .19 | 51 | 59 | 37 | 29.69 | 30.03 | 60 | 63 | WSW | NNE | Showery | Rain | Cloud. |
| 31 | | .08 | 41 | 50 | 36 | 30.14 | 30.43 | 61 | 57 | St. NE | St. NE | Fine | Showery with Hail | Fair |
| Apr | | | | | | | | | | | | | | |
| 1 | | | 42 | 50 | 40 | 30.35 | 30.28 | 57 | 60 | NNE | N | Cloudy | Fine | Fair |
| 2 | | | 42 | 48 | 38 | 30 33 | 30.39 | 63 | 56 | NE | NE | Showery | Fine | |
| 3 | | | 41 | 51 | 40 | 30.42 | 30.40 | 51 | 56 | NE | NNW | Fine | | Fair |
| 4 | | | 43 | 50 | 40 | 30.35 | 30.29 | 50 | 53 | NW | NW | Fair | Cloud. | |
| 5 | | | 42 | 53 | 38 | 30.17 | 30.01 | 56 | 57 | NW | NNW | Cloudy | | |
| 6 | ○ | .11 | 40 | 51 | 40 | 29.98 | 29.96 | 45 | 57 | NNW | N | Fine | Sho'ry | Fair |
| 7 | | | 41 | 53 | 39 | 30.00 | 30.05 | 51 | 55 | NNE | SE | Fair | Fine | Cloud. |
| 8 | | .19 | 40 | 49 | 37 | 30.07 | 30.10 | 57 | 60 | ENE | E | Fine | H.&R. | Fine |
| 9 | | | 39 | 48 | 38 | 30.01 | 30.00 | 60 | 60 | NE | NE | SnowHa. | Hail | Fine |
| 10 | | | 38 | 49 | 36 | 30.07 | 30.25 | 63 | 61 | ENE | NE | SnowHa. | H.&R. | Fair |
| 11 | | .43 | 38 | 50 | 37 | 29.97 | 29.99 | 55 | 57 | E var. | E var. | Fine | Sho'ry | Fine |
| 12 | | .22 | 40 | 51 | 40 | 29.80 | 29.84 | 68 | 65 | ESE | SE | Rain | | Fair |
| 13 | | | 41 | 49 | 39 | 29.72 | 29.96 | 70 | 68 | SSW | SW | Showery | Rain | Cloud. |
| 14 | ☽ | .18 | 41 | 50 | 40 | 30.00 | 30.02 | 63 | 60 | SE | SE | Foggy | Fair | Cloud. |
| 15 | | | 42 | 49 | 39 | 30.01 | 30.01 | 57 | 63 | ESE | ESE | Fair | | Rain |
| 16 | | .20 | 40 | 50 | 38 | 30.00 | 30.05 | 65 | 63 | SE | SSE | Showery | Fair | Fine |
| 17 | | | 41 | 52 | 41 | 29.89 | 29.77 | 71 | 75 | SE | SE | Foggy | Sho'ry | Cloud. |
| 18 | | | 48 | 55 | 43 | 29.76 | 29.70 | 71 | 71 | ENE | NE | Cloudy | | |
| 19 | | .15 | 49 | 58 | 45 | 29.71 | 29.74 | 68 | 70 | NNW | W | Fine | Thunder Lightn.&c | Rain |

The quantity of rain during the month of March, was 1.50 of an inch.

